# Detection of HPV RNA in Extracellular Vesicles from Neuroendocrine Cervical Cancer Cells

**DOI:** 10.3390/v14102226

**Published:** 2022-10-10

**Authors:** Faris Alkhilaiwi, Hang Yuan

**Affiliations:** 1 Department of Natural Products and Alternative Medicine, Faculty of Pharmacy, King Abdulaziz University, Jeddah 21589, Saudi Arabia; 2 Regenerative Medicine Unit, King Fahd Medical Research Center; King Abdulaziz University, Jeddah 22254, Saudi Arabia; 3 Department of Pathology, Georgetown University Medical School, Washington, DC 20057, USA

**Keywords:** human papillomavirus, infection, PCR, quantitation, viral load, HPV-6

## Abstract

**Background:** Neuroendocrine carcinoma of the cervix (NECC) is an aggressive and rare type of cervical cancer. The five-year overall survival is low at 30% and there is no standardized therapy based on controlled trials for this type of tumour. Most are locally advanced or metastasized at the time of the diagnosis. Extracellular vesicles (EVs) could be a carrier of viral DNA/RNA, given their vital role in cellular communication. The content of EV derived from NECC cells has not been investigated due to the lack of cell line, and it is not known whether they contain human papillomaviruses (HPV) DNA/RNA or not. **Methods:** The presence of viral E7 DNA/RNA in EVs purified from a culture of a recently established NECC cell line, GUMC-395, was evaluated by using droplet digital polymerase chain reaction (ddPCR). These EVs were characterized using nanoparticle tracking analysis (NTA) for size distribution, transmission electron microscopy (TEM) for morphology, Western blot for CD63, and bioanalyser for RNA quantity and quality. **Results:** HPV16 viral-RNA, but not DNA, was detected in EVs from GUMC-395 using ddPCR. NTA identified EVs with a mean diameter of 105.0 nm, TEM confirmed normal morphological shape and size, and Western blot analysis confirmed the presence of EV-associated proteins CD63. The EVs were found to be enriched with small RNAs using a bioanalyser. **Conclusions:** HPV16 RNA is found in EVs from a neuroendocrine cervical cancer and could be involved in the pathogenesis of the disease and used as a diagnostic biomarker.

## 1. Introduction

High-risk human papillomaviruses (hrHPV) are proven carcinogens in the oropharynx, penis, cervix, vagina, vulva, and anus [1]. Cervical cancer alone contributes 3.4% of deaths caused by different types of cancer globally. More than 341,831 women died in 2020 due to cervical cancer [2]. Of cervical cancer cases, 99.7% are associated with hrHPV, mainly HPV16 and HPV18 [3]. Nearly 10% of cervical cancers are adenocarcinomas, whereas 80% are squamous cell carcinomas and a small fraction are neuroendocrine carcinomas. Unlike most cervical cancers, cervical neuroendocrine cancer has a poor prognosis due to its highly aggressive malignancy paradoxically in early stages, and it is regularly undetected by Pap smears owing to the high endocervical location, the normal overlying epithelium, and its downward growth pattern [4]. EVs have been considered vital for developing numerous kinds of pathologies, including cancer and neurodegenerative in recent years. To date, there are hundreds of articles showing the involvement of EVs in cancer development [5,6]. Recently, some studies have assessed the role of EVs in cancer advancement since these EVs are involved in cellular communication through the transport of their cargos from donor cells to receptor cells and the subsequent changes of cellular processes, allowing tumour advancement [7]. The EV content includes proteins, RNA, and DNA; this content can be altered by HPV infection. The oncoproteins E6 and E7 increased the levels of miR-21 and miR-146 in EVs isolated from cervicovaginal lavages of women with HPV-positive cervical cancer [8,9,10]. Moreover, these viral oncoproteins could produce changes in the EV content of proteins such as surviving and p53 and RNAs such as lncRNAs [11,12]. Additionally, mRNA encoding for HPV16 E6/E7 was detected in EV-derived SiHa cells and from human foreskin keratinocytes (HFK) cells that were transduced with HPV16 E6/E7 [9]. The transfer of viral DNA or genomic DNA in other types of cancer into exosomes has been proven [13,14]. The detection of viral DNA and RNA in EVs is useful to increase our understanding about the mechanism of cancer progression and as a tumour biomarker. In addition, infection and other factors in the microenvironment can influence the transformation and tumour progression. This microenvironment is composed of vascular, endothelial, immune, and all other cells in tissue. Therefore, the main purpose of this study was to evaluate the presence of HPV DNA and RNA in EVs derived from HPV-16-positive cell cultures of neuroendocrine cervical cancer GUMC-395, where the viral genome is integrated in the human genome.

## 2. Materials and Methods

### 2.1. Cell Culture and Isolation of Evs

GUMC-395 cells (p3) were cultured in a collagen type I-coated flask using F medium supplemented with 10% fetal bovine serum, 0.125 ng/mL epidermal growth factor (Invitrogen, Carlsbad, CA, USA), 25 ng/mL hydrocortisone (Sigma–Aldrich, Burlington, MA, USA), 5 μg/mL insulin (Sigma-Aldrich), 0.1 nM cholera toxin (Sigma-Aldrich), in addition to 10 μM Y-27632 (Sigma-Aldrich). HFK cells (p4) were grown in serum-free medium (Life Technologies, Carlsbad, CA, USA). All cells were maintained in a humidified incubator with 5% CO_2_ at 37 °C [15,16]. Dneasy Blood & Tissue Kit (Qiagen, Hilden, Germany) was used to isolate DNA from cells. HFK and GUMC-395 DNA preparation were subjected, using HPV16 primers, to polymerase chain reaction (PCR) analysis according to the protocol previously described [15]. Primer sequences: HPV16, 5-TTATGAGCAATTAAATGACAGCTCAG-3, and 5′-TGAGAACAGATGGGGCACACAAT-3′. EVs were isolated from conditioned media (25 mL collected from 6 million cells) after being centrifuged at 1000× *g* for 10 min then filtered using 0.22 nm filter. The resulting supernatant was used to isolate EVs using ExoQuick reagent (SBI, product #EXOQ5A, lot #140624-001) following the manufacturer’s protocol and resuspended in 50 µL of PBS.

### 2.2. Nanoparticle Tracking Analysis (NTA)

Using ZetaView PMX-420 Analyser (Particle Metrix, Meerbusch, Germany) NTA measurements were performed. The simultaneously built-in lasers, with an excitation wavelength of 405 nm and 640 nm, were used for fluorescence detection, whereas the 488 nm laser was used for all scatter applications. Analysis of videos that were taken at 30 frames per second for concentration and size using the ZetaView software version 8.05.10 (Particle Metrix, Meerbusch, Germany) with anti-bleach technology.

### 2.3. Transmission Electron Microscopy (TEM)

The EV pellet was resuspended in PBS/2% paraformaldehyde, then 2 µL were plated on carbon-coated grids (Ted Pella Inc., Redding, CA, USA). After allowing for adsorption, the grids were transferred to 1.5% glutaraldehyde for 10 min after being washed many times in PBS. Then, the grids were transferred to a uranyl-oxalate solution for 5 min. They were dried and observed at 80 kV in a transmission electron microscope equipped with CCD camera for image acquisition (HitachiH-7600, Tokyo, Japan).

### 2.4. Western Blot Analysis

Proteins were isolated and Western blot analysis was performed as previously described [17], with anti-CD63 antibody at a dilution of 1:1000 (EXOAB-CD63A-1, SBI), anti-Calnexin antibody at a dilution of 1:1000 (SC-11397, SANTA CRUZ BIOTECHNOLOGY), and goat anti-Rabbit HRP secondary antibody at 1:20,000 dilution.

### 2.5. RNA Integrity and Quality by Bioanalyser

The extraction of total RNA from GUMC-395 and EVs using an miRNeasy Kit (Qiagen, Hilden, Germany) was conducted as described previously [18]. The integrity and quantity of total and small RNAs were assessed using an RNA 6000 Pico Kit (Agilent Technologies, Santa Clara, CA, USA), and Agilent 2100 Bioanalyser (Agilent) using a Small RNA Kit (Agilent Technologies, Santa Clara, CA, USA).

### 2.6. Droplet Digital PCR

For the HPV16 detection assay, the QX200 Droplet Generator (Bio-Rad, Hercules, CA, USA) was used to generate the droplets as per the manufacturer’s directions. Reactions were partitioned into a median of approximately 15,000 droplets per well using the QX200 droplet generator. All reactions were performed on a QX200 ddPCR system (Bio-Rad, Hercules, CA, USA) in technical duplicates. The HPV16 E7 Tagman assay used was forward primer 5′-(CAAGTGTGACTCTACGCTTCGG)-3′, (reverse primer) 5′-( GTGGCCCATTAACAGGTCTTCCAA)-3′and probe FAM-5′- TGCGTACAAAGCACACACGTAGACATTCGT -3′- MGBNFQ.

### 2.7. Statistical Analysis

Two independent assay measurements of total DNA and RNA concentration were made for the HPV E7 assays. Each assay measurement comprised data from four replicates of ddPCR wells for E7 and two replicates for the no-template control.

## 3. Results

### 3.1. General Characterisation of Evs Derived from GUMC-395

**Figure 1:**
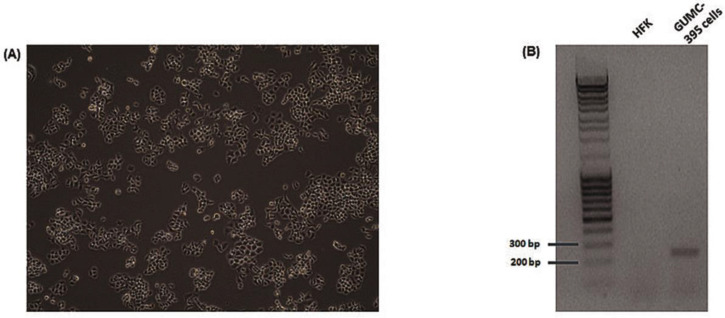
**Phase-contrast photograph and HPV detection of GUMC-395.** (**A**) Cell morphology of GUMC-395 growing on collagen-coated flasks. (**B**) HPV16 detection in GUMC-395 but not in HFK by PCR assay. DNAs were isolated from GUMC-395 or HFKs. PCR reactions were performed with an HPV-16-specific primer set. PCR products were separated on an agarose gel.

**Figure 2:**
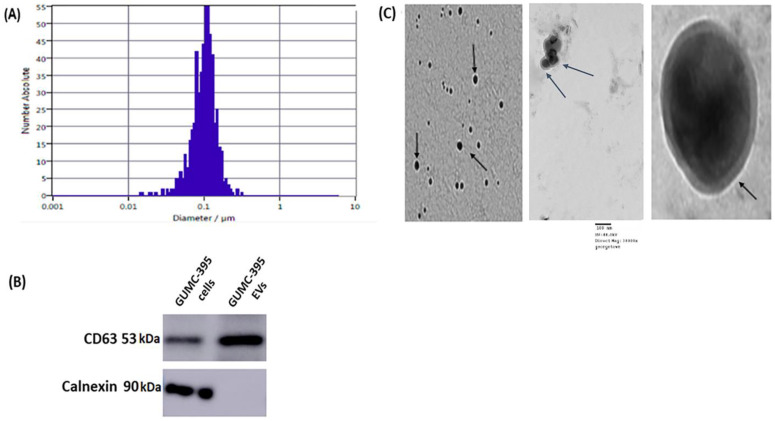
**Characterization of Evs from GUMC-395.** (**A**) The size distribution profile of GUMC-395-derived EVs. (**B**) Representative Western blot of three (*n* = 3) independent experiments for EV marker CD63 and lacking Calnexin (original blots are presented in Supplementary Figure S1). (**C**) Representative images of EVs (black arrows point toward EVs) from GUMC-395 by TEM. Scale bars = 100 nm.

GUMC-395 cells were grown on a collagen-coated flask in the presence of Y-27632 (Figure 1A) and were tested for HPV16 (Figure 1B). The culture supernatants of neuroendocrine cervical cancer cells GUMC-395 were processed to obtain purified EVs, and the data are shown in (Figure 2). Using ZetaView NTA, vesicles isolated by SBI were found to have a mean size distribution of 105.0 ± 35 nm (Figure 2A). Western blot analysis was used to detect proteins that are expressed in the EVs. We detected CD63 as the EVs’ enriched marker but not Calnexin, which is a control protein localized in the endoplasmic reticulum and does not exist in EVs (Figure 2B). Then, we confirmed the size and morphology of EVs via TEM. The result established heterogeneous sizes and round shape morphology of EVs, which was the same as the previously recognized morphology of EVs (Figure 2C) [19].

### 3.2. GUMC-395 EVs Are Enriched with Small RNAs and miRNAs

**Figure 3:**
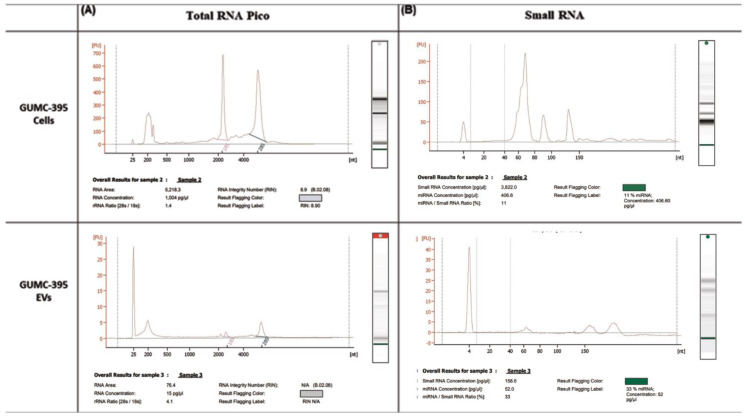
**Characterisation of RNA extracted from GUMC-395 cells and their EVs.** RNA quality was evaluated using (**A**) RNA 6000 Pico kit and (**B**) small RNA kit from Agilent Bioanalyser for GUMC-395 cells and their EVs were extracted using SBI isolation techniques. The *x*-axis is the size of the RNA, measured in nucleotides (nt), and the *y*-axis represents fluorescence.

Total RNA was extracted from GUMC-395 cells and GUMC-395 cells derived from EVs and were analyzed using the RNA 6000 Pico kit and the small RNA kit from Agilent bioanalyser. While there were ribosomal RNAs in GUMC-395 cells (Figure 3A, upper panel), there was no or very little detection of ribosomal RNA (18S and 28S) in the EV fraction (Figure 3A, lower panel), indicating pure fraction of EVs. On the other hand, small RNAs (<200 nucleotides) were present and enriched with 33% of small RNA are miRNA in EVs fraction (Figure 3B, lower panel) versus 11% in cellular RNA (Figure 3B, upper panel).

### 3.3. Detection and Quantification of HPV DNA in GUMC-395 Evs by ddPCR

DNA was isolated from GUMC-395 cells and EVs derived from GUMC-395 cells, whereas cDNA was made from purified RNA from GUMC-395 cells, and EVs derived from GUMC-395 cells were tested using ddPCR. Serial dilution of both GUMC-395 cells genomic and EVs DNA as well as the cDNA were used to detect and accurately measure the concentration of E7 copies per μL. Dnase treatment was included to make sure the DNA was inside the EV, not outside it.

**Figure 4:**
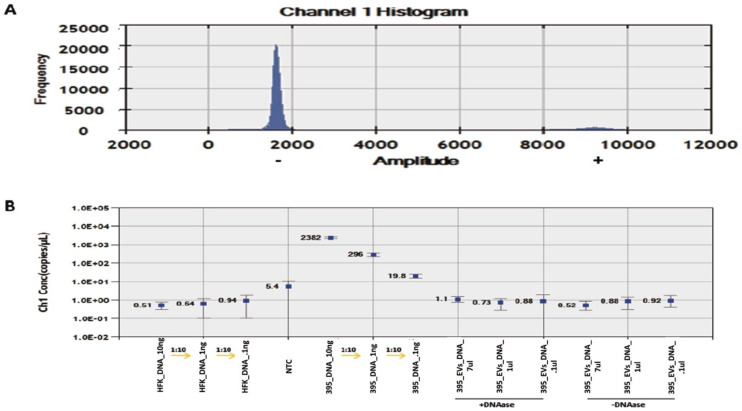
**ddPCR detection of HPV E7 DNA in serially diluted HFK, GUMC-395, and GUMC-395 EVs.** (**A**) Histogram results for droplet separation based on HPV E7 assays. Positive droplets were observed at an amplitude of about 9000, whereas negative droplets were observed at an amplitude of about 1800. (**B**) The concentrations (copies/μL) of the HPV E7 as processed by QuantaSoft. The concentration and Poisson confidence intervals for each “merged” well were computed using the QuantaSoft Software. Concentrations (copies/μL) represent the measurement of HPV E7 DNA in merged wells for each sample. Error bars indicate the Poisson 95% confidence intervals for each measurement. HFK = Human Foreskin Keratinocytes (−HPV); GUMC-395(+HPV); NTC = No Template Control.

The results showed a linear correlation between GUMC-395 cellular DNA concentration and HPV16 E7 copies/μL, as expected; however, EV samples showed no correlation and no positive fluorescence signals similar to the negative control for HFK (Figure 4), indicating no detection of viral DNA inside the EVs.

**Figure 5:**
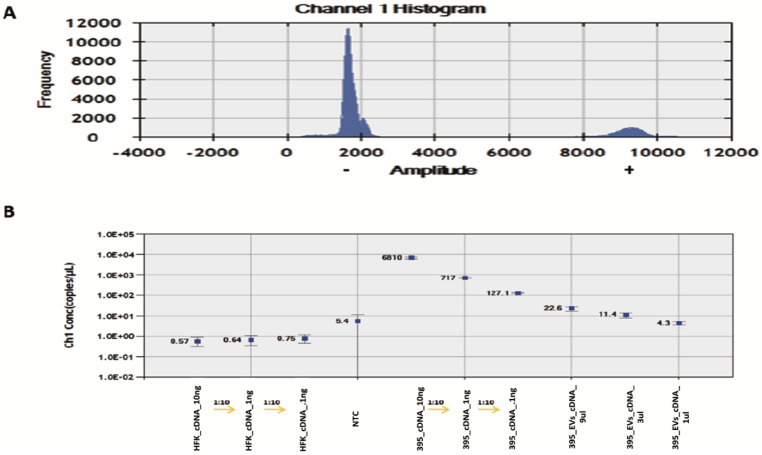
**ddPCR detection of HPV E7 cDNA in serially diluted HFK, GUMC-395, and GUMC-395 EVs.** (**A**) Histogram results for droplet separation based on HPV E7 assays. Positive droplets were observed at an amplitude of about 9000, whereas negative droplets were observed at an amplitude of about 1800. (**B**) The concentrations (copies/μL) of the HPV E7 as processed by QuantaSoft. The concentration and Poisson confidence intervals for each “merged” well were computed using the QuantaSoft Software. Concentrations (copies/μL) represent the measurement of HPV E7 cDNA in merged wells for each sample. Error bars indicate the Poisson 95% confidence intervals for each measurement. HFK = Human Foreskin Keratinocytes (−HPV); GUMC-395(+HPV); NTC = No Template Control.

On the other hand, the similar linear correlation between GUMC-395 cDNA concentration and HPV16 E7 copies/μL were as expected. Moreover, EV samples showed positive fluorescence signals and linear correlation (Figure 5), indicating detection of viral mRNA E7 inside the EVs.

## 4. Discussion

Neuroendocrine cervical cancer is inadequately understood because of the absence of a suitable in vitro model that would help understanding of the disease’s etiology. Yuanet al. used the conditional reprogramming technology to establish the first cell line named GUMC-395 from neuroendocrine cervical cancer, which is positive with HPV16 [15]. GUMC-395 cells derived from EVs were isolated and viral RNA was detected using droplet digital PCR. On the other hand, no viral DNA was detected.

By using PCR and NGS data, Mata-Rocha et al. have shown the presence of E6–E7 DNA-HPV fragments in EVs isolated from HeLa cells’ condition media and cervical exudate samples (HPV18) irrespective of the integration status in the cell [20]. Furthermore, Nguyen et al. have detected HPV DNA/RNA in EVs isolated from plasma samples from oropharyngeal squamous cell carcinoma patients. However, detection of cf-DNA/RNA had statistically higher sensitivity compared to EV-DNA/RNA for circulating tumour HPV DNA and HPV RNA [21]. On the other hand, Jeannot et al. have detected viral DNA in serum samples (cfDNA sample) from cervical cancer patients with HPV-associated invasive cancers using ddPCR, and its level is correlated to tumour dynamics. Nevertheless, they did not detect positive signals in patients with HPV-associated high grade cervical intraepithelial neoplasia [22]. EVs have been recognized as vital signaling mediators in various pathophysiological and physiological processes through transfer of bioactive molecules such as proteins, miRNA, and mRNAs between cells [23]. Thus, proper functional studies should be conducted to investigate the potential transfer of the viral RNA in EVs to neighboring cells. Thorough functional studies should be conducted next since EVs have been confirmed as mediators for the progression of various types of cancers. Therefore, the inhibition of exosome release in neuroendocrine cervical cancer may slow the progression of the disease, providing potential therapeutic targets [24].

Our study is limited by the limited number of cell lines generated (one cell line); however, rare cancers such as neuroendocrine cervical cancer are very difficult to obtain. Additionally, blood samples are lacking to test whether ddPCR is sensitive enough to detect the viral RNA in biological fluid.

Our results confirm the presence of HPV RNA in EV with a potential clinical utility in the form of valid tests for the detection of circulating tumour HPV RNA, therefore providing a tool for diagnosing HPV-neuroendocrine cancer. Specifically, since such cancer shows false negative results on the Pap smear, it can be challenging to diagnose. Droplet digital PCR is an efficient technique for viral RNA quantification and detection and, therefore, can be used for neuroendocrine cervical cancer diagnosis.

## Data Availability

Not applicable.

## Supplementary Materials

The following supporting information can be downloaded at: https://www.mdpi.com/article/10.3390/v14102226/s1, Figure S1: Full blot for (A) CD63 and (B) Calnexin.

## References

[1] Bucchi D., Stracci F., Buonora N., Masanotti G. (2016). Human papillomavirus and gastrointestinal cancer: A review. World J. Gastroenterol..

[2] Sung H., Ferlay J., Siegel R.L., Laversanne M., Soerjomataram I., Jemal A., Bray F. (2021). Global Cancer Statistics 2020: GLOBOCAN Estimates of Incidence and Mortality Worldwide for 36 Cancers in 185 Countries. CA Cancer J. Clin..

[3] Walboomers J.M., Jacobs M.V., Manos M.M., Bosch F.X., Kummer J.A., Shah K.V., Snijders P.J., Peto J., Meijer C.J., Muñoz N. (1999). Human papillomavirus is a necessary cause of invasive cervical cancer worldwide. J. Pathol..

[4] McCusker M.E., Coté T.R., Clegg L.X., Tavassoli F.J. (2003). Endocrine tumors of the uterine cervix: Incidence, demographics, and survival with comparison to squamous cell carcinoma. Gynecol. Oncol..

[5] Bebelman M.P., Smit M.J., Pegtel D.M., Baglio S.R. (2018). Biogenesis and function of extracellular vesicles in cancer. Pharmacol. Ther..

[6] Minciacchi V.R., Freeman M.R., Di Vizio D. (2015). Extracellular vesicles in cancer: Exosomes, microvesicles and the emerging role of large oncosomes. Semin. Cell Dev. Biol..

[7] Guenat D., Hermetet F., Prétet J.L., Mougin C. (2017). Exosomes and Other Extracellular Vesicles in HPV Transmission and Carcinogenesis. Viruses.

[8] Harden M.E., Munger K. (2017). Human papillomavirus 16 E6 and E7 oncoprotein expression alters microRNA expression in extracellular vesicles. Virology.

[9] Chiantore M.V., Mangino G., Iuliano M., Zangrillo M.S., De Lillis I., Vaccari G., Accardi R., Tommasino M., Columba Cabezas S., Federico M. (2016). Human papillomavirus E6 and E7 oncoproteins affect the expression of cancer-related microRNAs: Additional evidence in HPV-induced tumorigenesis. J. Cancer Res. Clin. Oncol..

[10] Liu J., Sun H., Wang X., Yu Q., Li S., Yu X., Gong W. (2014). Increased exosomal microRNA-21 and microRNA-146a levels in the cervicovaginal lavage specimens of patients with cervical cancer. Int. J. Mol. Sci..

[11] Zhang J., Liu S.C., Luo X.H., Tao G.X., Guan M., Yuan H., Hu D.K. (2016). Exosomal Long Noncoding RNAs are Differentially Expressed in the Cervicovaginal Lavage Samples of Cervical Cancer Patients. J. Clin. Lab. Anal..

[12] Honegger A., Leitz J., Bulkescher J., Hoppe-Seyler K., Hoppe-Seyler F. (2013). Silencing of human papillomavirus (HPV) E6/E7 oncogene expression affects both the contents and the amounts of extracellular microvesicles released from HPV-positive cancer cells. Int. J. Cancer.

[13] Thakur B.K., Zhang H., Becker A., Matei I., Huang Y., Costa-Silva B., Zheng Y., Hoshino A., Brazier H., Xiang J. (2014). Double-stranded DNA in exosomes: A novel biomarker in cancer detection. Cell Res..

[14] Sanada T., Hirata Y., Naito Y., Yamamoto N., Kikkawa Y., Ishida Y., Yamasaki C., Tateno C., Ochiya T., Kohara M. (2016). Transmission of HBV DNA Mediated by Ceramide-Triggered Extracellular Vesicles. Cell Mol. Gastroenterol. Hepatol..

[15] Yuan H., Krawczyk E., Blancato J., Albanese C., Zhou D., Wang N., Paul S., Alkhilaiwi F., Palechor-Ceron N., Dakic A. (2017). HPV positive neuroendocrine cervical cancer cells are dependent on Myc but not E6/E7 viral oncogenes. Sci. Rep..

[16] Liu X., Krawczyk E., Suprynowicz F.A., Palechor-Ceron N., Yuan H., Dakic A., Simic V., Zheng Y.L., Sripadhan P., Chen C. (2017). Conditional reprogramming and long-term expansion of normal and tumor cells from human biospecimens. Nat. Protoc..

[17] Perut F., Roncuzzi L., Zini N., Massa A., Baldini N. (2019). Extracellular Nanovesicles Secreted by Human Osteosarcoma Cells Promote Angiogenesis. Cancers.

[18] Izumi H., Tsuda M., Sato Y., Kosaka N., Ochiya T., Iwamoto H., Namba K., Takeda Y. (2015). Bovine milk exosomes contain microRNA and mRNA and are taken up by human macrophages. J. Dairy Sci..

[19] Kruger S., Abd Elmageed Z.Y., Hawke D.H., Wörner P.M., Jansen D.A., Abdel-Mageed A.B., Alt E.U., Izadpanah R. (2014). Molecular characterization of exosome-like vesicles from breast cancer cells. BMC Cancer.

[20] Mata-Rocha M., Rodríguez-Hernández R.M., Chávez-Olmos P., Garrido E., Robles-Vázquez C., Aguilar-Ruiz S., Torres-Aguilar H., González-Torres C., Gaytan-Cervantes J., Mejía-Aranguré J.M. (2020). Presence of HPV DNA in extracellular vesicles from HeLa cells and cervical samples. Enferm. Infecc. Microbiol. Clin..

[21] Nguyen B., Meehan K., Pereira M.R., Mirzai B., Lim S.H., Leslie C., Clark M., Sader C., Friedland P., Lindsay A. (2020). A comparative study of extracellular vesicle-associated and cell-free DNA and RNA for HPV detection in oropharyngeal squamous cell carcinoma. Sci. Rep..

[22] Jeannot E., Becette V., Campitelli M., Calméjane M.A., Lappartient E., Ruff E., Saada S., Holmes A., Bellet D., Sastre-Garau X. (2016). Circulating human papillomavirus DNA detected using droplet digital PCR in the serum of patients diagnosed with early stage human papillomavirus-associated invasive carcinoma. J. Pathol. Clin. Res..

[23] Valadi H., Ekström K., Bossios A., Sjöstrand M., Lee J.J., Lötvall J.O. (2007). Exosome-mediated transfer of mRNAs and microRNAs is a novel mechanism of genetic exchange between cells. Nat. Cell Biol..

[24] Zhang H., Lu J., Liu J., Zhang G., Lu A. (2020). Advances in the discovery of exosome inhibitors in cancer. J. Enzyme Inhib. Med. Chem..

