# Supplementary figures and images for: Detection of HPV RNA in Extracellular Vesicles from Neuroendocrine Cervical Cancer Cells
Source: Viruses. 2022 Oct 10;14(10):2226. doi: 10.3390/v14102226 (PMC9606890; doi:10.3390/v14102226)

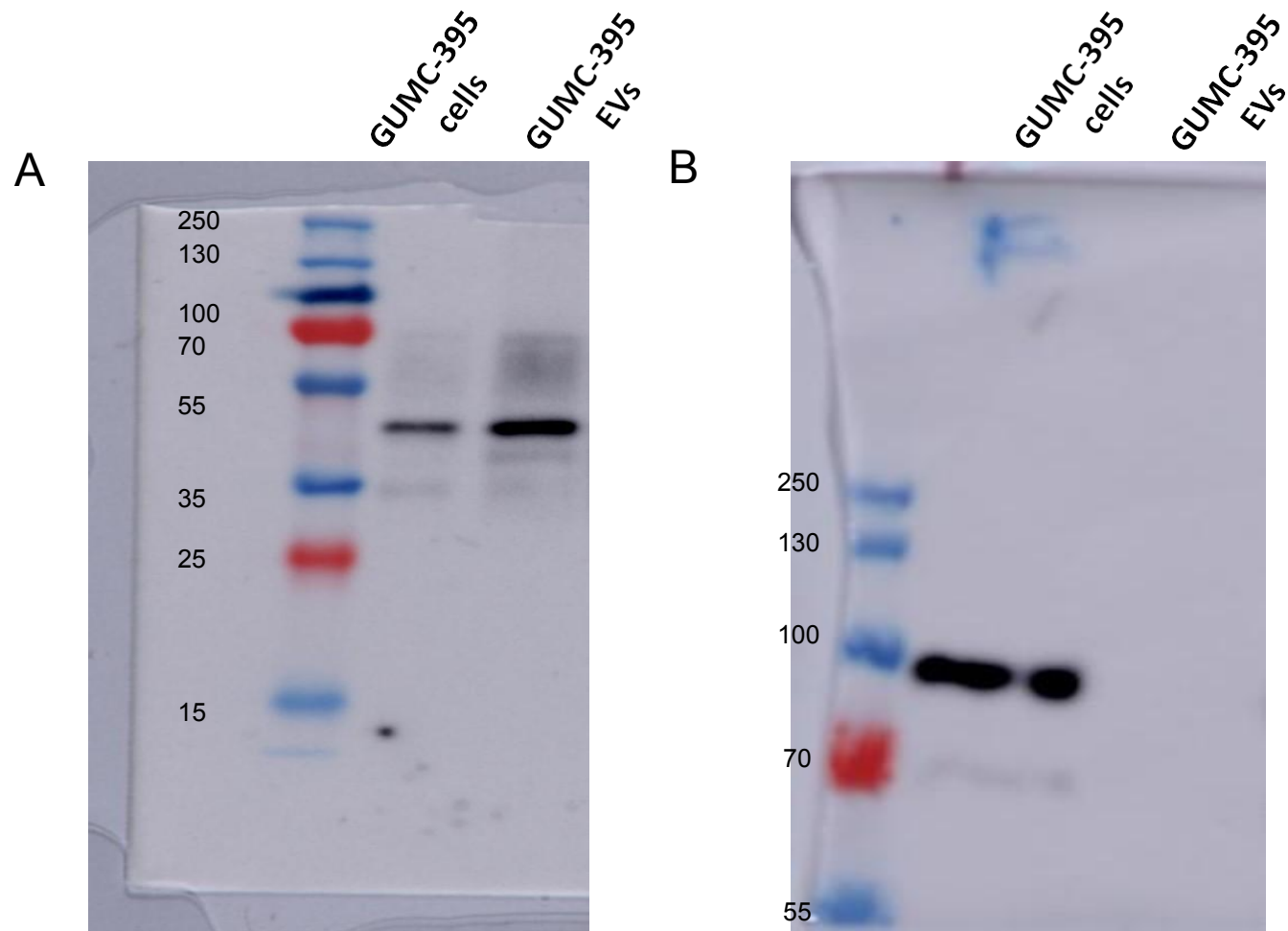

**Supplementary Figure S1. Full blot for (A) CD63 and (B) Calnexin.**

Supplement: Supplementary file 1 [file viruses-14-02226-s001.zip › viruses-1900750-supplementary.pdf]
